# Resilience in Health Care: The Role of Telemedicine and e-Health Enabling Adaptive Strategies in Sleep Medicine Practice During the COVID-19 Pandemic

**DOI:** 10.1177/26924366251378098

**Published:** 2025-09-23

**Authors:** Mithri R. Junna, Amy Glasgow, Timothy I. Morgenthaler

**Affiliations:** ^1^ Division of Pulmonary and Critical Care Medicine, Center for Sleep Medicine, Mayo Clinic, Rochester, Minnesota, USA.; ^2^ Division of Sleep Neurology, Department of Neurology, Mayo Clinic, Rochester, Minnesota, USA.; ^3^ Robert D. and Patricia E. Kern Center for the Science of Health Care Delivery, Mayo Clinic, Rochester, Minnesota, USA.

**Keywords:** resilience strategies, health care delivery, crisis management, sleep medicine

## Abstract

**Background:**

The COVID-19 pandemic brought profound changes to health care systems worldwide. This study examines the adaptive responses of a sleep medicine practice that successfully pivoted to telemedicine, remote diagnostics, and agile operations, resulting in sustained growth and improved patient accessibility.

**Methods:**

We analyzed organizational and cultural elements enabling responsiveness, including telemedicine infrastructure, adaptive leadership, quality-focused processes, and effective communication. Objective outcomes were assessed by analyzing patient demographics, telemedicine visits, and sleep testing patterns during three phases: pre-pandemic (June 1, 2018 to March 8, 2020), acute COVID-19 (March 9, 2020 to April 19, 2020), and post-acute COVID-19 (April 20, 2020 to December 31, 2021).

**Results:**

Across 105,199 encounters, monthly sleep medicine visits and testing declined ∼50% during acute COVID-19, but rebounded to surpass baseline in the post-acute phase. Virtual visits increased significantly, replacing many face-to-face encounters. Home sleep apnea test use rose sharply, driven by disposable WatchPAT tests, while polysomnography use decreased. Testing volumes increased overall post-acute compared with pre-pandemic. Patients served during acute and post-acute phases were younger, more often female, non-White, and postgraduate-educated (all *p* < 0.001). Nonlocal patients increased during the post-acute phase, reflecting telemedicine expansion.

**Conclusions:**

Proactive telemedicine investments, adaptive leadership, quality-focused processes, and effective communication enabled this practice to adapt successfully during the pandemic. These resilience strategies provide a model for navigating future health care challenges, underscoring telemedicine and e-health’s critical roles in maintaining care delivery during crises.

## Introduction

The concept of resilience emphasizes an organization’s capacity to anticipate, adapt, and evolve in response to disruptions.^1–3^ Our practice’s pre-pandemic investment in telemedicine and disposable home sleep apnea tests (HSATs), training in team-based quality improvement methodology, and optimization of our electronic health record (EHR) laid the foundation for a rapid shift when COVID-19 altered the health care landscape. As highlighted by Kim et al., organizational resilience emerges from a multi-level approach involving leadership, operational processes, and individual adaptability.^4^ In particular, resilience in health care is complex and often anchored in quality management principles that drive reliability and adaptability across the organization.^5^

The COVID-19 pandemic caused unprecedented disruption to health care delivery worldwide, necessitating rapid adaptations across all medical specialties.^6^ In the early months of the pandemic, as stay-at-home orders and social distancing measures were implemented, health care systems faced the dual challenge of maintaining essential care while safeguarding patients and providers from viral transmission.^7^

Sleep medicine, a field deeply reliant on in-person diagnostic and therapeutic practices up until that time, experienced particularly significant upheavals that underscored the critical need for resilience in health care. Prior to the COVID-19 pandemic, most of the clinical care required face-to-face (F2F) visits, in-lab polysomnography (PSG) testing, and follow-up visits relying on the ability to download data from positive airway pressure (PAP) therapy devices in-person. In March and April of 2020, the American Academy of Sleep Medicine issued guidance recommending the postponement of in-lab PSGs and administration of PAP therapy except in urgent cases. Nearly 90% of sleep centers in the United States reported halting or significantly reducing in-laboratory sleep testing, with HSAT volumes also declining by 60%.^8,9^

Outside the United States, sleep medicine practices faced similar disruptions. In Europe, a study of 40 centers reported in-lab PSGs dropping from 92.5% pre-pandemic to 20% during, with PAP therapy during PSG falling to 17.5%. Still, 36 of 39 centers maintained follow-ups for sleep-disordered breathing via telehealth.^10^ In Korea, 85% of 20 surveyed centers reduced PSGs during the pandemic’s third wave, with varying changes in PAP practices.^11^ In India, 72% of 75 sleep physicians stopped all sleep testing during lockdowns, though 20% continued HSATs and 8% performed limited in-lab tests.^12^ In Iran, referrals showed fewer PSGs and obstructive sleep apnea (OSA) diagnoses, but higher rates of insomnia and other sleep issues.^8^

The pandemic nearly halted in-person sleep medicine services worldwide. Despite this, the demand for quality sleep care increased due to heightened anxiety, social instability, medication shortages, limited exposure to natural light, and disrupted routines, leading to increased insomnia and other sleep complaints.^9,13^ This highlighted the necessity of sleep medicine services, even with severe constraints on testing and therapy.

Providers had to rapidly innovate and adapt to meet escalating demand with limited capacity, showcasing the importance of resilience. This paper discusses how our sleep medicine practice overcame disruptions, restored pre-pandemic service levels, and gained new capabilities through a resilient response. Telemedicine and e-health played a crucial role in continuing care.^14^ These experiences provide valuable lessons on building robust systems capable of handling large-scale disruptions in health care.

## Methods

We provide an overview of the organizational and cultural elements that enabled responsiveness and key actions undertaken during critical pandemic phases. To assess outcomes, we analyzed patient demographics, telemedicine visits, and sleep testing patterns during three phases: pre-pandemic (PrePan), acute COVID-19 (AcuteCOV), and post-acute COVID-19 (PostAcuteCOV). The retrospective review included all patients aged 18+ seen by sleep medicine providers at Mayo Clinic sites in Rochester, Arizona, Florida, and the Mayo Clinic Health System. All data were queried via the EHR. The analysis evaluated how adaptive strategies sustained patient care and enabled service expansion. Statistical analyses (Shapiro–Wilk test for normality and chi-square tests for categorical variables) assessed shifts in patient engagement and operational modes across phases.

The Mayo Clinic Institutional Review Board waived review of this study because of its principal focus on systems of health care delivery.

### Organizational and cultural readiness

Mayo Clinic’s culture is defined by its patient-centered ethos, where “the needs of the patient come first” drives all decisions.^15^ It emphasizes collaborative teamwork, physician leadership, commitment to excellence, and strong institutional values like respect, compassion, and innovation. The integrated practice model fosters consistency and resource sharing across campuses, while its nonprofit mission ensures reinvestment into research, education, and patient care. A focus on employee well-being, organizational longevity, and accessibility of leadership promotes a supportive and resilient work environment.^16,17^ Staff pride in Mayo Clinic’s global reputation reinforces a shared commitment to delivering high-quality care.

Mayo Clinic integrates education and continuous improvement into its culture through initiatives like the Quality Academy, which supports employees from onboarding to leadership development.^18^ The Academy emphasizes disciplines such as process mapping, Lean principles, swim lane diagrams, and failure modes and effects analyses. Continuous improvement and operational excellence are woven into clinical and research efforts at every level.^19^ Continuous improvement is central to clinical and research efforts. The Center for Sleep Medicine has long aligned quality initiatives with clinical innovation, fostering a culture of learning and adaptability. This approach reflects Braithwaite et al.’s findings on embedding quality and evidence-based improvements in organizational culture to enhance resilience and patient safety during crises.^20^

### Actions

Enabled by this culture and capability as a foundation, our actions and their outcomes can be examined across three distinct time periods for this study: (1) from June 1, 2018, to March 8, 2020 (current EHR started); (2) from March 9, 2020, to April 19, 2020 (stay at home orders were in place); and (3) from April 20, 2020, to December 31, 2021 (based upon American Academy of Sleep Medicine guidance). These periods correspond to PrePan, AcuteCOV (during which we were developing our virtual capacity and under instructions to limit F2F encounters), and PostAcuteCOV (wherein we had established infrastructure to support telephone, video, or F2F encounters and to conduct HSATs or in-laboratory PSG testing).

Prior to the COVID-19 pandemic, we had already initiated several actions based on our belief that enhancing telemedicine and remote diagnostic sleep medicine services was essential for better patient care. This primarily involved preparing for video visit capabilities and improving HSAT capabilities.

Eighteen months before the onset of COVID-19, our center equipped every exam room with cameras, speakers, and microphones to facilitate natural video visits for providers. Initially, our experience with video visits was minimal, but we set an arbitrary goal of having 5% of our visits conducted via video by the end of the year to ensure all providers and support staff gained the necessary exposure and experience.

To expand video visit capabilities, we leveraged Mayo Clinic’s centralized telemedicine efforts under the Center for Connected Care, which provided a comprehensive infrastructure to support telemedicine services across the organization.^21^ A detailed operational plan was developed to delineate roles clearly: the Center for Connected Care managed core administrative operations, while clinical departments retained ownership of clinical services. The plan focused on long-term success through standardized products, implementation services, and staffing models. We had also begun programming efforts to ensure remote access to cloud-based PAP use data so that the physical presence of these devices would not be needed to assess use and efficacy.^21^

In September of 2019, at the World Sleep Conference in Vancouver, Canada, one of our authors (T.I.M.) saw an early version of a disposable HSAT that used the patient’s mobile phone and a cloud-based system to enable completely remote testing and review in evaluating patients with a high pre-test probability for having OSA.^22^ Upon return from the conference, work began immediately to create the EHR integration, training, and operational processes and to obtain the needed cyber-security features to begin using this test. A series of Plan-Do-Study-Act (quality improvement cycle) (PDSA) cycles led to improvement of the processes such that by January 2020, we could easily order and use such tests with high operational efficiency, low data loss, and high diagnostic utility.

Our initial investment in telemedicine infrastructure, remote HSAT kits, cloud-based PAP adherence and efficacy data, and integration into our EHR system enabled rapid scalability when in-person care was restricted. This proactive approach to infrastructure readiness and expanding capabilities reflects the “anticipatory resilience” described by Madani and Parast, where preparatory investments reduce cognitive load during a crisis, facilitating swift adjustments and service continuity.^5^

### Team Agility and Problem-Solving

The onset of the AcuteCOV period was abrupt, and our organizational response was rapid. Using the technological infrastructure described above, we quickly pivoted to enable providers to work from the safety of their home using the same digital tools developed for use in the office. Workers were issued laptops and other needed equipment from a central supply station and returned home. From there, daily agile meetings, replacing monthly operational sessions, became a cornerstone for real-time problem-solving. Morning check-ins and afternoon debriefs allowed for dynamic responses to emerging challenges, aligning with Karreinen et al.’s findings on the importance of daily adaptability in resilient health care settings.^1^ Teams across all levels could apply Lean methodologies, like PDSA cycles, to streamline processes, enabling efficient patient care amidst operational flux.

Communication played a vital role in sustaining team morale and alignment, particularly during the AcuteCOV period. Regular, structured check-ins facilitated transparent sharing of challenges, solutions, and affirmations, reinforcing a supportive culture during a period of heightened stress. The emphasis on inclusive communication and support parallels findings from Kim et al., where decentralized decision-making and leader accessibility were critical in maintaining operational coherence and individual resilience during crises.^4^

Most of the care during the AcuteCOV period was delivered by providers working from home, with essential staff working in the center, and only limited diagnostic testing taking place within the laboratory at first.

While many patients with common sleep disorders like OSA could be treated with telemedicine, HSAT, and automatic PAP therapy, this approach did not fit sicker patients needing advanced testing. During AcuteCOV, we had to ensure safe titration of PAP therapy in our sleep lab during PSG. Our technologists and clinicians collaborated to develop innovative solutions, including enhanced infection control and downstream filters on PAP masks. These measures ensured continued patient care and technologist safety, allowing a gradual return of sleep laboratory capabilities to serve our patients.

### Analysis

To analyze the results of our efforts across the three time periods, we gathered information regarding total number of visits completed with further categorization into telehealth (video and telephone) or F2F visits and total number of HSAT and PSG tests completed with further identification of the age, gender, race, educational level, and zip code of patients undergoing these visits and diagnostic tests.

We compared the total number and kinds of visits and tests completed during each of the three timeframes detailed above to examine differences. All continuous data distributions were evaluated for normality using the Shapiro–Wilk test. Data are summarized as mean ± SD when normally distributed, or as median and interquartile range [median (Q1, Q3)] when nonnormally distributed. Categorical variables are reported as *n* (%). We determined whether the COVID-19 pandemic led to changes in visit or testing patterns. A secondary analysis was undertaken to see if any observed changes in visit or testing patterns were associated with age, gender, race, education level, or domicile locations. Rates of visits or tests were compared using chi-square or unpaired *t*-test methods as appropriate. The values of *p* < 0.05 were considered significant. Statistical analysis was conducted using SAS version 9.4 (SAS Institute Inc, Cary, NC).

### Outcomes

**FIG. 1.**
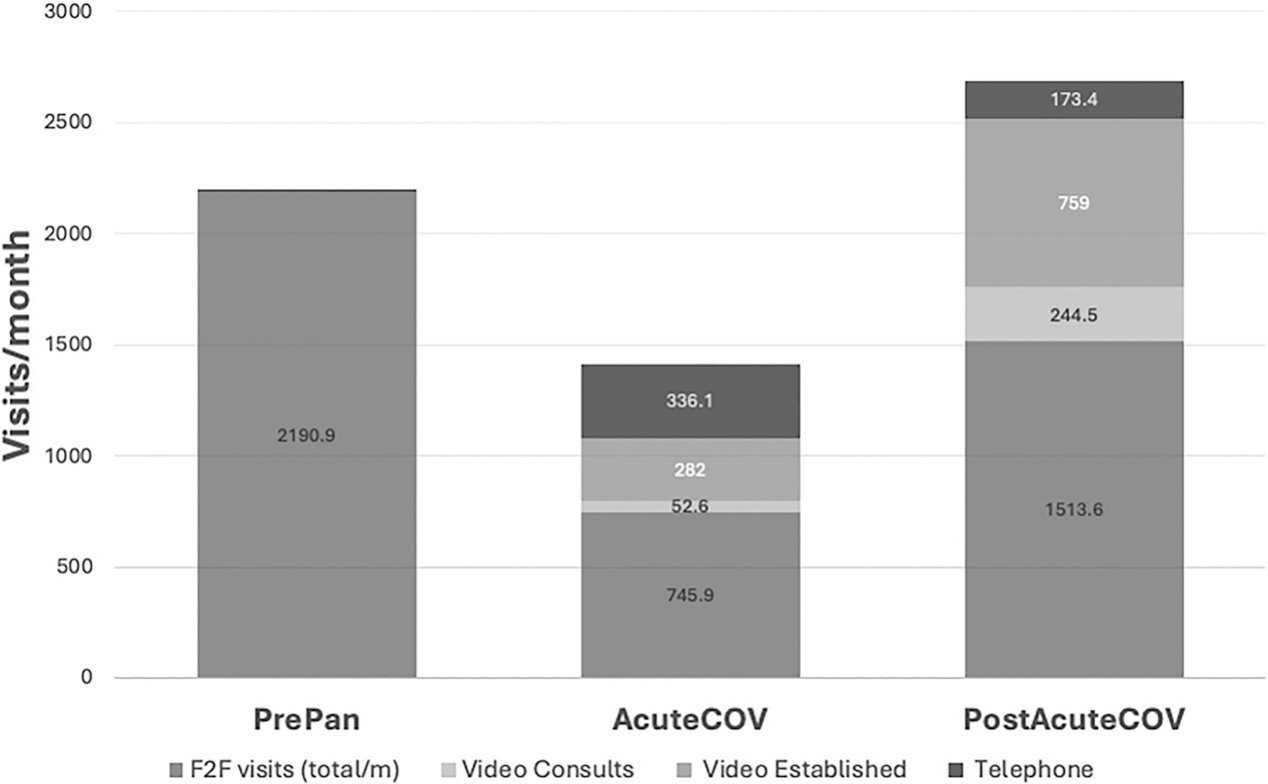
Visit types across pandemic periods (visits/month). Face-to-face (F2F) visits include consults and established visits. The greatest changes seen across the different periods were the number of telemedicine visits, which included video consults, video established visits, and a smaller number of telephone-only visits. In the acute COVID-19 (AcuteCOV) period, telemedicine visits comprised 47% of all visits. In the post-acute COVID-19 (PostAcuteCOV) period, telemedicine visits comprised 44% of all visits. While very uncommon in the pre-pandemic (PrePan) period, by the PostAcuteCOV period, the number of video established patient visits had grown to be of similar frequency (759.0/month) to F2F-established patient visits (894.7/month).

**FIG. 2.**
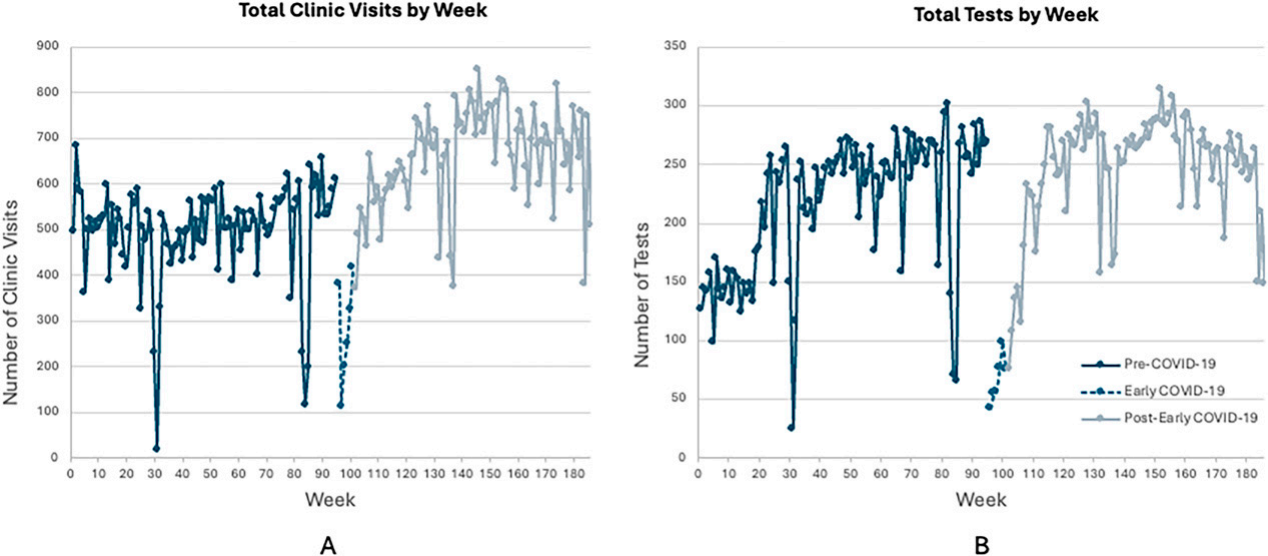
Visits and tests by week. The total clinic visits **(A)** and tests **(B)** by week increased over the 180 weeks under observation of this study, with very sharp declines occurring coincident with the orders to shelter at home. The visits and tests recovered quickly as capacity for both telehealth and face-to-face (F2F) visits was restored. Both the clinic visits/week and tests/week declined slightly after reopening of capabilities, illustrative of pent-up demand during the shelter at home orders.

During the combined three periods studied, we observed a total of 105,199 encounters. As anticipated, the number of visits per month significantly declined during the AcuteCOV period, followed by an increase surpassing baseline levels in the PostAcuteCOV period (Table 1, Figures 1 and 2A). While virtual visits (via telephone or video) were infrequent before the pandemic, their utilization rose markedly during both the AcuteCOV and especially the PostAcuteCOV periods. Conversely, F2F visits decreased compared with the PrePan period. Telephone visits were more prevalent during the AcuteCOV phase, but saw a notable reduction in the PostAcuteCOV phase, with video visit volume increasing significantly.

**Table 1.** Average Visit Volumes by Period (Encounter Types/Month), All Patient Types

Encounter type	I. PrePan June 1, 2018, through March 8, 2020	II. AcuteCOV March 9, 2020, through April 19, 2020	III. PostAcuteCOV April 20, 2020, through December 31, 2021	*p* Overall	*p* I vs II	*p* I vs III
Total visits/mo	2194.7	1416.5	2690.6	<0.0001	<0.0001	<0.0001
F2F visits (total/mo)	2190.9	745.9	1513.6	<0.0001	<0.0001	<0.0001
F2F New	67.0	21.8	46.3	<0.0001	<0.0001	<0.0001
F2F Con	848.8	302.3	572.6	<0.0001	<0.0001	<0.0001
F2F Established	1275.1	421.8	894.7	<0.0001	<0.0001	<0.0001
Televideo (total/mo)	3.8	670.7	1177.0	<0.0001	<0.0001	<0.0001
Video New	0.4	13.5	44.3	<0.0001	<0.0001	<0.0001
Video Con	0	39.1	200.2	<0.0001	<0.0001	<0.0001
Video Established	3.4	282.0	759.0	<0.0001	<0.0001	<0.0001
Telephone	0.05	336.1	173.4	<0.0001	<0.0001	<0.0001
Sleep tests/month	944.9	495.8	1052.4	<0.0001	<0.0001	<0.0001
PSG any	635.0	178.5	505.9	<0.0001	<0.0001	<0.0001
PSG w PAP	260.1	59.8	178.5	<0.0001	<0.0001	<0.0001
HSAT any	309.9	317.3	546.5	<0.0001	0.6384	<0.0001
HSAT Type III	104.3	10.3	38.4	<0.0001	<0.0001	<0.0001
HSAT WatchPAT (not mail-out)	194.4	194.0	292.0	<0.0001	0.9754	<0.0001
HSAT WatchPAT (mail-out disposable)	11.9	112.9	216.1	<0.0001	<0.0001	<0.0001

AcuteCOV, acute COVID-19; F2F, face-to-face; HSAT, home sleep apnea test; PAP, positive airway pressure; PostAcuteCOV, post-acute COVID-19; PrePan, pre-pandemic; PSG, polysomnography.

**FIG. 3.**
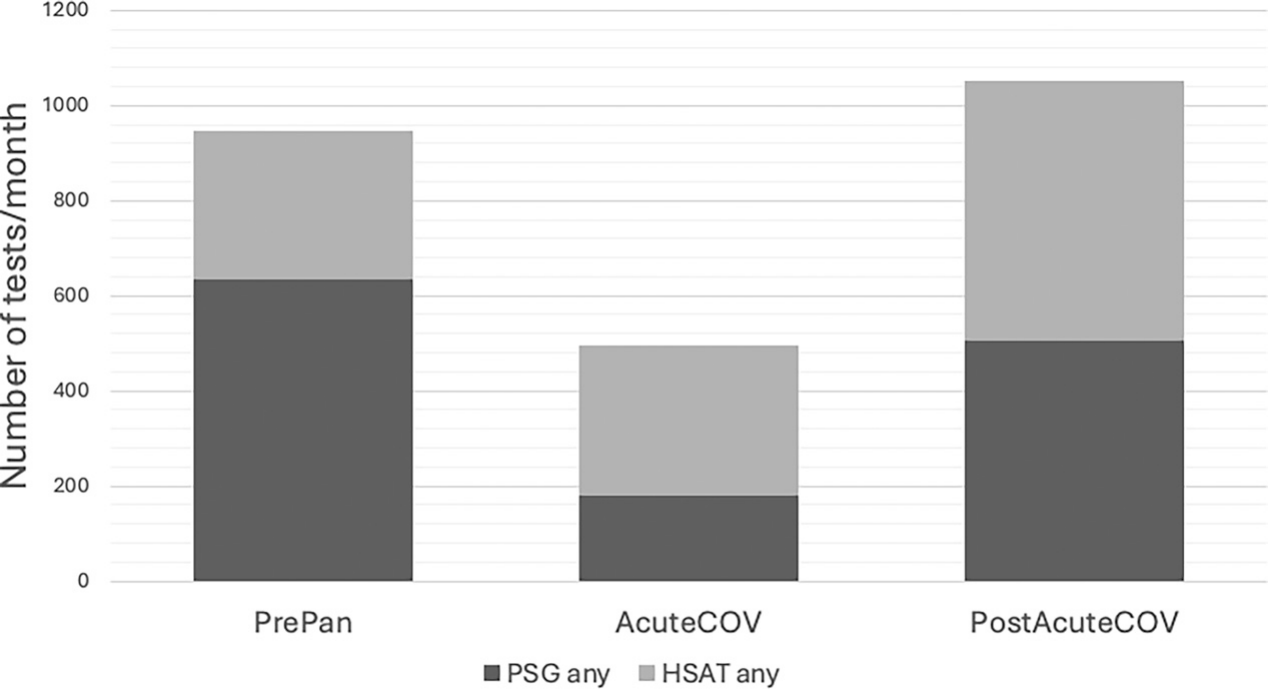
Sleep tests across pandemic periods. During the pre-pandemic (PrePan) period, home sleep apnea tests (HSATs) comprised 33% of all sleep tests performed, with a comparable number of WatchPAT and Type-III HSATs and very few mailout disposable WatchPAT tests. During the acute COVID-19 (AcuteCOV) period, in-lab polysomnography (PSG) testing declined sharply, with very few tests involving positive airway pressure (PAP) titration due to infection concerns. During the post-acute COVID-19 (PostAcuteCOV) period, 52% of all sleep studies performed were HSATs. Most tests were performed for suspected obstructive sleep apnea (OSA), and because fewer PAP titrations were performed, more patients were prescribed auto-titrating PAP devices.

Sleep testing also declined by nearly 50% during the AcuteCOV period, with a sharp shift from PSG toward HSAT (Table 1, Figures 2B and 3). In our center, we moved quickly toward using disposable WatchPAT tests (WatchPAT One™ by Itamar Medical, Inc.), which could be mailed to the patient and data gathered via the internet, thereby avoiding infection risks and travel. The overall testing volume increased when comparing PrePan to PostAcuteCOV periods. PSG use declined in the PostAcuteCOV phase compared with PrePan, while the use of HSAT—especially the disposable WatchPAT test—increased.

The demographic characteristics of the patients we served via the various appointment types differed across the three periods (Table 2). Compared with the PrePan phase, patients served in the AcuteCOV and PostAcuteCOV phases tended to be younger, with a higher proportion of females, higher level of education, and a higher proportion of non-White patients (all *p* < 0.001). The home address of the patients being served also varied. During the AcuteCOV phase, local regional domiciles increased from 55.7% to 56.9%, but in the PostAcuteCOV phase, we found ourselves serving a more distant population (52.1% local region). This was perhaps caused by the increase in telemedicine services.

**Table 2.** Patient Characteristics for Clinic Encounters (Excluding Testing) at Different Periods

	I. PrePan June 1, 2018, through March 8, 2020	II. AcuteCOV March 9, 2020, through April 19, 2020	III. PostAcuteCOV April 20, 2020, through December 31, 2021	*p* Overall	*p* I vs II	*p* I vs III
N encounters	46,813	1,884	56,502			
Age (mean ± SD, median (IQR)	61.0 ± 15.1 63 (52, 72)	59.4 ± 15.2 62 (49, 70)	60.6 ± 15.4 63 (51, 72)	<0.0001	<0.0001	0.0008
% Female	18947 (40.5%)	764 (40.6%)	24236 (42.9%)	<0.0001	0.9779	<0.0001
Educational levels				<0.0001	0.0354	<0.0001
Did not graduate high school	874 (2.5%)	28 (1.7%)	909 (1.7%)			
High school graduate	6,279 (17.8%)	273 (16.3%)	8,657 (16.6%)			
Associate degree	10,226 (29.0%)	511 (30.6%)	15,190 (29.2%)			
Four-year degree	9,254 (26.3%)	470 (28.1%)	14,846 (28.5%)			
Postgraduate education	8586 (24.4%)	390 (23.3%)	12460 (23.9%)			
Missing	11,594	212	4,440			
Domicile in local geographical location	26084 (55.7%)	1072 (56.9%)	29412 (52.1%)	<0.0001	0.3117	<0.0001
Race				0.0002	0.8096	<0.0001
Hispanic	1,889 (4.0%)	75 (4.0%)	2,435 (4.3%)			
White	41,378 (88.4%)	1,657 (88.0%)	49,866 (88.3%)			
Black	1,466 (3.1%)	66 (3.5%)	1,735 (3.1%)			
Asian	783 (1.7%)	37 (2.0%)	1,092 (1.9%)			
Other	685 (1.5%)	26 (1.4%)	770 (1.4%)			
Unknown	632 (1.4%)	23 (1.2%)	604 (1.1%)			

IQR, interquartile range; SD, standard deviation.

Testing patterns changed significantly across different periods. We assessed if these changes varied by demographic factors (Table 3). Patients tested during AcuteCOV were younger than those PrePan, but age differences were not significant PostAcuteCOV. The proportion of females was slightly lower in AcuteCOV, but significantly higher afterward. In AcuteCOV, more patients had postgraduate education than PrePan. Local patient proportions increased during both AcuteCOV and PostAcuteCOV periods compared with PrePan (*p* < 0.01). Additionally, there was an increase in non-White patients compared with PrePan.

**Table 3.** Patient Characteristics for Testing at Different Periods

	I. PrePan June 1, 2018, through March 8, 2020	II. AcuteCOV March 9, 2020, through April 19, 2020	III. PostAcuteCOV April 20, 2020, through December 31, 2021	*p* Overall	*p* I vs II	*p* I vs III
*N* encounters	20179	672	20909			
Age (mean ± SD, median [IQR])	57.5 (15.5) 59 (47, 69)	55.5 (15.1) 57 (44, 67)	57.7 (15.5) 60 (47, 69)	0.0005	0.0003	0.3386
% Female	8547 (42.4%)	276 (41.1%)	9234 (44.2%)	0.0006	0.5073	0.0002
Educational levels				<0.0001	0.1324	<0.0001
Did not graduate high school	317 (2.8%)	13 (2.4%)	374 (2.0%)			
High school graduate	2104 (18.4%)	76 (14.1%)	3373 (18.3%)			
Associate degree	3586 (31.4%)	181 (33.5%)	5792 (31.4%)			
Four-year degree	2762 (24.2%)	137 (25.4%)	4932 (26.7%)			
Postgraduate education	2654 (23.25)	133 (24.6%)	3989 (21.6%)			
Missing	8756	132	2449			
Domicile in local geographical location	9471 (46.9%)	355 (52.8%)	10085 (48.2%)	0.0008	0.0026	0.0085
Race				<0.0001	0.1110	<0.0001
Hispanic	798 (4.0%)	33 (4.9%)	1003 (4.8%)			
White	17906 (88.7%)	578 (86.0%)	18317 (87.6%)			
Black	622 (3.1%)	28 (4.2%)	741 (3.5%)			
Asian	348 (1.7%)	17 (2.5%)	405 (1.9%)			
Other	251 (1.2%)	5 (0.7%)	233 (1.1%)			
Unknown	254 (1.3%)	11 (1.6%)	210 (1.0%)			

IQR, interquartile range; SD, standard deviation.

Most testing was performed for suspected OSA, but other diagnostic codes were linked to sleep visits and testing in approximately 13% (Table 4). Although there was a statistically significant change in the prevalence of different diagnostic codes linked to the tests, most of the differences seemed to be driven by a reduced variety of linked diagnoses with tests performed during the AcuteCOV period compared with PrePan or PostAcuteCOV periods.

**Table 4.** Diagnostic Codes Linked to Sleep Tests at Time of Billing

	I. PrePan June 1, 2018, through March 8, 2020	II. AcuteCOV March 9, 2020, through April 19, 2020	III. PostAcuteCOV April 20, 2020, through December 31, 2021	*p* Overall	*p* I vs II	*p* I vs III
Indication				<0.0001	<0.0001	<0.0001
Sleep apnea	17401 (86.2%)	602 (89.6%)	18217 (87.1%)			
Insomnia	93 (0.5%)	1 (0.1%)	104 (0.5%)			
Hypersomnia	332 (1.6%)	8 (1.2%)	210 (1.0%)			
Parasomnia	216 (1.1%)	1 (0.1%)	157 (0.8%)			
Neuro	144 (0.7%)	3 (0.4%)	119 (0.8%)			
Multiple	260 (1.3%)	6 (0.9%)	282 (1.3%)			
Other	1733 (8.6%)	51 (7.6%)	1820 (8.7%)			

## Discussion

We believe our practice’s evolution underscores the power of proactive infrastructure, agile operational models, and quality improvement skills to achieve resilience in health care. We demonstrated that preemptive planning and a quality-centered culture can mitigate crisis impacts, positioning health care organizations to not just survive, but thrive. As shown by Schulman et al., focusing on network resilience—where reliability and adaptability are embedded at every level—yields benefits that extend beyond individual organizations to positively impact patient outcomes and access across health care networks.^2^

The high transmissibility and mortality rates associated with the onset of the COVID-19 pandemic necessitated the implementation of “stay-at-home” orders and significant modifications to nonemergent and ambulatory clinical services in early March 2020. This study analyzed the impact on service provision during the acute phase of the pandemic, a period characterized by the unavailability or transition to telemedicine for most elective and ambulatory services. In the field of sleep medicine, facility-based sleep testing was temporarily suspended, requiring time to establish telemedicine services and alternative testing methods. For our organization, this did not take a great deal of time—approximately 45 days to restore volume, if not kinds, of service.^23^

As anticipated, patient visits declined sharply during the AcuteCOV phase, with total visits decreasing by 35.4%, and 47% of services transitioning to televideo consultations.^23^ Our facility resumed more regular staffing of outpatient clinics on April 20, incorporating measures such as mandatory masking and eye protection, social distancing, frequent surface sanitization with antiviral solutions, and limiting the number of visitors accompanying patients.

During the recovery from severe restrictions, we observed a rebound in total patient visit volumes, eventually surpassing PrePan levels. This “overshoot” likely represented accumulated demand for chronic disease management services coupled with ongoing needs for new evaluations. It may also reflect our organization’s rapid return to capability to provide service when others could not.

However, during the COVID-19 pandemic, there was an increase in sleep complaints such as insomnia and a general rise in the prevalence of obesity, and it is possible that more individuals developed risk factors for and were referred for evaluation of OSA during this observation period. Insomnia, in particular, was commonly observed in other previously mentioned sleep medicine practices at New York University Langone Health and in practices across India.^9,13^

There was a marked increase in the use of telemedicine services in our sleep medicine practices, paralleling other reports of increased telemedicine utilization. Those who were younger, female, more educated, and non-White tended to utilize telemedicine services more. Horrell et al. reported similar findings in their survey based investigation of telemedicine during COVID-19, specifically the increased uptake of telemedicine by younger, female, and better educated patients.^24^ Those who are younger and more educated may be more comfortable with or have more access to the use of technology required for telemedicine services.^25^ Women generally are reportedly more likely than men to search for a provider online, to communicate with a health care provider online, and when working from home, tend to shoulder more family care responsibilities than men.^25^ All of these factors may favor females as accessing telemedicine services more frequently than men.

Many of the changes in visit and testing patterns accompanied marked changes in regulatory and insurance permissiveness for use of telemedicine as part of the Department of Health and Human Services Federal Public Health Emergency for COVID-19, declared under Section 319 of the Public Health Service Act, which expired on May 11, 2023. In some ways, the observed changes seem to suggest increased accessibility for patients (women and those further away from our medical center) who could not previously access our sleep medicine services. Our center began monitoring patient experience metrics specific to telemedicine services in the time intervals studied and found generally high levels of satisfaction.

## Conclusion

The COVID-19 pandemic catalyzed significant changes across health care, underscoring the need for resilience strategies that ensure continuity and adaptability. Our sleep medicine practice, grounded in agile infrastructure, comprehensive quality initiatives, and effective team collaboration, provides a model for resilience that can guide other health care organizations in preparing for future disruptions. By focusing on quality improvement capabilities and adaptive processes, health care organizations can maintain high standards of patient care and emerge stronger from periods of crisis. We are hopeful that this experience has taught us how to successfully navigate potential future challenges to our practice.

## Abbreviations Used

AASM: American Academy of Sleep Medicine
AcuteCOV: Acute COVID-19 period
CON: Consult
COVID-19: Coronavirus disease 2019
EHR: Electronic health record
EST: Established
F2F: Face-to-Face
HSAT: Home sleep apnea test
IQR: Interquartile range
OSA: Obstructive sleep apnea
PAP: Positive airway pressure
PDSA: Plan-Do-Study-Act (quality improvement cycle)
PostAcuteCOV: Post-acute COVID-19 period
PrePan: Pre-pandemic
PSG: Polysomnography
SD: Standard deviation
